# Caries status among 6-year-old children born with cleft lip and palate in Norway: a nationwide study

**DOI:** 10.3389/froh.2025.1669481

**Published:** 2025-09-19

**Authors:** O. E. Osman, M. A. Yassin, R. Bjering, P. K. Saele, I. M. T. Korsnes, M. O. B. Hagbø, Å. Sivertsen, S. A. Lie

**Affiliations:** 1 Center for Translational Oral Research, Department of Clinical Dentistry, Medical Faculty, University of Bergen, Bergen, Norway; 2 Department of Plastic Surgery, Section for Cleft Lip and Palate, Rikshospitalet, Oslo University Hospital, Oslo, Norway; 3 Oral Health Centre of Expertise/Western Norway, Bergen, Norway; 4 Department of Plastic Surgery, Haukeland University Hospital, Bergen, Norway; 5 The Norwegian National Network for Arthroplasty and Hip Fractures, Haukeland University Hospital, Bergen, Norway

**Keywords:** cleft lip, cleft palate, dental caries, congenital deformities, Norway

## Abstract

**Background:**

Orofacial cleft (OFC) is the most common congenital malformation affecting the craniofacial area. A major challenge in managing children with OFC is their increased susceptibility to dental caries. This study aims to explore the association between dental caries and various potential variables, including OFC types, sex, and the presence of additional congenital deformities.

**Methods:**

This study includes 6-year-old children with OFC registered in the Norwegian Registry of Cleft Lip and Palate. Children were classified into two main groups: cleft lip and/or palate (CL ± P)—including cleft lip only (CLO) and cleft lip and palate (CLP)—and cleft palate only (CPO). The association between dental caries and OFC types, sex, and additional congenital deformities was analyzed using chi-square tests and logistic regression, reporting *p*-values and odds ratios (OR) with 95% confidence intervals (CI).

**Results:**

Among 551 children with OFC (343 boys and 208 girls), 37% had CLP, 36% had CPO, and 27% had CLO. Dental caries experience was found among 99 (18%) children (66 males and 33 females): 39 had CPO, 37 had CLP, and 23 had CLO. No association between dental caries and OFC type was found (*p* = 0.627). However, a significant association was found between dental caries and the presence of additional congenital deformities (OR = 2.10, 95% CI: 1.30–3.39, *p* = 0.002).

**Conclusion:**

Boys are more commonly affected by OFC than girls. Children with OFC and additional congenital deformities are more susceptible to caries, indicating the need for more targeted and individualized preventive oral health strategies.

## 1 Introduction

Cleft lip (CL) and cleft palate (CP) are congenital malformations where incomplete fusion of the embryonic facial processes occurs. These may present as complete or incomplete unilateral cleft lip and/or palate (UCL ± P) or bilateral cleft lip and/or palate (BCL ± P) defects (1, 2). They represent as the most common congenital defects in the head and neck region (2–6), with a worldwide prevalence of 1 in 700 (7). In Norway, the incidence is estimated to be 2 in 1,000 (8). A difference in the prevalence of clefts between boys and girls has been demonstrated (9, 10). There is a considerable percentage of children with orofacial cleft (OFC) who have other congenital deformities (9), including central nervous system deformities, congenital heart diseases, and syndromes, as reported in 14.5% (11) and 20% (12) of cases. The congenital deformities or syndromes accompanying OFC may affect both management and treatment strategies for this population (11). Children with OFC usually need highly comprehensive management from a specialized multidisciplinary team to meet their different dental, medical, psychological, and behavioral requirements throughout life (13). This applies to daily challenges, including hearing, speech, feeding, appearance concerns, dental hygiene, and caries prevention (14). Dental caries or tooth decay is a complex illness that is impacted by several variables, including food, oral hygiene habits, and the availability of dental treatment (15). In addition, dental caries can affect daily performance due to the pain associated with it (16). Furthermore, caries is considered a global public health problem and a threat to children's oral health (17). The malformed anatomy of the oral cavity of children with OFC can result in difficulties in maintaining optimal dental health, which may result in dental caries (18). A higher prevalence (19) and experience (20) of dental caries have been reported among this population (21). The prevalence of dental caries varies depending on the type of OFC. For example, children with bilateral cleft lip and palate (CLP) have been reported to have a higher prevalence of dental caries than that of children with unilateral cleft lip and palate (17). Nevertheless, OFC condition not only affects the appearance of the children but also affects the shape and continuity of the maxillary arch and the number and shape of the teeth in the oral cavity (18). Children with OFC undergo repeated management and treatment through their childhood and adulthood, including orthodontic interventions, soft tissue surgical interventions, bone transplants, and dental implants (22, 23). These children are at risk of maxillary growth restriction, dental crowding, and permanent teeth missing (tooth agenesis) (24). Thus, primary teeth maintenance in children with OFC is crucial to the future successful eruption of the permanent teeth (25). Primary teeth also help with eating, speaking, and keeping space for permanent teeth (25). As children with OFC often miss some permanent teeth (tooth agenesis) (26), losing primary teeth too early could lead to later space loss and arch disruption during the permanent dentition (25). Primary teeth, besides being functionally important, are also an important factor for the success of psychological support by enabling a healthy smile on the children's faces (27). Researchers and clinicians often use the decay, missing, and filled primary teeth (DMFT) scoring system, which is commonly used for scoring tooth decay. This system does not always apply to all datasets. In some studies (e.g., large-scale datasets or registry-based data), it may be more practical just to record whether a child has caries or not, as a binary (yes/no) outcome. This approach has been used in some large-scale studies (28). Furthermore, the majority of current evidence on caries in children with OFC comes from studies focusing on permanent dentition. Less information on caries in primary (milk) teeth has been reported (29). In contrast, this study focuses on caries experience in primary dentition. Available data from the Norwegian Registry of Cleft Lip and Palate (NRCLP) was used. This registry enables population-level research with minimal selection bias. This distinctive potential is rarely observed in international research, which often studies limited samples from single hospitals or dental clinics. Existing data about caries diagnosis within the registry were based on a dichotomized DMFT scoring system as either no for no caries experience (DMFT = 0) or yes (DMFT ≥ 1). This diagnosis was obtained through clinical inspection and on radiographs [orthopantomogram (OPG) or periapical x-rays]. The present study aims to assess the experience of dental caries in primary teeth among 6-year-old children with OFC in Norway. Furthermore, we aim to study the association between dental caries and different types of OFC, accounting for the sex of the child and other additional congenital deformities.

## 2 Materials and methods

The data for this study were obtained from the NRCLP, which has registered newborns since 2011. The registry collects data on all children registered and treated for CLP in Norway. The registry's compliance rate is 90%. The present study was designed to investigate the association between dental caries and different types of OFC, sex, and other additional congenital deformities among OFC children in Norway. The materials include complete data on 6-year-old children born with OFC in Norway and registered in the registry (*n* = 551). In Norway, the treatment of all children with OFC is centralized at two central centers: Oslo University Hospital (in Oslo) and Haukeland University Hospital (in Bergen). Data in the registry were recorded by a plastic surgeon at the time of all cleft surgeries, in addition to follow-up visits at ages of 4 and 16 years. Specialists in orthodontics record data of children at ages 6, 10, and 16 years. Information about dental caries is recorded in the registry as (yes/no/do not know). This recording pattern limits the quantification of the severity of the lesions. However, it is practical for public health surveillance since “yes” will be equivalent to DMFT ≥ 1. Information about additional congenital anomalies besides the OFC was registered similarly (yes/no/uncertain).

Children were classified into two main cleft types: “CL ± P, which includes cleft lip only (CLO) and CLP” and “cleft palate only (CPO)” (30). Furthermore, the laterality of the cleft was registered as UCL ± P or BCL ± P. At age 6, children undergo oral examination, including registration of caries status. We included all 551 children with information about the type of cleft, caries, and clinical data. Children aged below 6 were excluded.

The data extracted from the registry database included sex, type of OFC, associated additional congenital deformities, syndromes, and dental caries experiences. Dental caries experience was considered the primary outcome and dependent variable in the analysis. Type of OFC was considered the primary independent variable (i.e., independent variable), while sex, syndromes, and the other congenital deformities, in addition to OFC, were considered secondary (or confounding) variables.

Informed consent was obtained from participants' parents or legal guardians at the time of enrollment in the registry. The study was approved by the Regional Committee for Medical and Health Research Ethics, Western Norway (REK West: 598456).

### Statistical analysis

Characteristics of categorical data were presented using percentages and frequencies with contingency tables to assess the associations between the variables. A chi-square test was used to assess whether associations in the contingency tables were statistically significant. Binary logistic regression models, reporting odds ratios (OR) with 95% confidence interval (95% CI), were applied for adjusted analyses. Statistical analysis was conducted using IBM SPSS Statistics for Windows, Version 29 (Armonk, NY, USA). *p* < 0.05 was considered statistically significant.

## 3 Results

The study included 551 6-year-old children born and diagnosed with OFC. Of this sample, 343 (62%) were boys, and 208 (38%) were girls, with a boy-to-girl ratio of 1.6:1. A total of 208 (37%) had CLP, 197 (36%) had CPO, and 146 (27%) had CLO (Table 1). The percentages of boys among the CLP, CLO, and CPO groups were as follows: 76%, 64%, and 47%, respectively. The boy-to-girl ratio was 2.4:1 among CL ± P children and 0.87:1 among CPO children (Table 1). A total of 72 (13%) of the children had bilateral cleft lip and/or palate (BCL ± P). In the BCL ± P group, 57 (79%) were boys and 15 (21%) were girls. A total of 281(51%) children had cleft alveolus, with a boy-to-girl ratio of 2.5:1 (Table 1). There was a statistically significant association between sex and the cleft type (*p* < 0.001). We found 120 (22%) children with congenital deformities in addition to OFC. Among these children, 68 (57%) were boys and 52 (43%) were girls (Table 1). Facial congenital deformities included ear tag (1%), pitting under the lower lip (2%), small chin (6%), ears (2%), eyes (3%), and other facial features (5%). Furthermore, some children with OFC had congenital heart disease (5%), muscle deformities (2%), central nervous system deformities (2%), dysmorphism (9%), and psychometric inadequacy (14%) (Table 1). A total of 78 (14%) children with OFC had syndromic conditions, of whom 41 (53%) were boys and 37 (47%) were girls (Table 1). From the comparable group of children in the general population, 18.2% had caries experience. Of the total children with OFC, 99 children (18%) had caries experience (Table 2), which was not significantly different from the comparable group from the general population (*p* = 0.891) (Table 2). Of these children with OFC and dental caries, 66 (67%) were boys and 33 (33%) were girls. A total of 23 (23%) had CLO, 37 (37%) had CLP, and 39 (40%) had CPO (Table 3). Nine of them (9%) had BCL ± P, and 48 (48%) had cleft alveolus. The association between dental caries experience and OFC types was not statistically significant (*p* = 0.627) (Table 3). Sixteen children had syndromic conditions, and 33 (33%) had congenital deformities other than OFC. A statistically significant association was found between dental caries and congenital deformities other than OFC (OR = 2.10, 95% CI: 1.30–3.39, *p* = 0.002) (Table 3).

**Table 1:** Frequencies and percentages of cleft type, syndromic children, and children with other congenital deformities stratified by children's sex.

Sub-group	Boys	%	Girls	%	Total	Boys:Girls
CPO	92	47	105	53	197	0.87:1
CLP	158	76	50	24	208	3.0:1
Right side CLP	32	71	13	29	45	2.5:1
Left side CLP	76	76	24	24	100	3.2:1
Bilateral CLP	50	79	13	21	63	3.8:1
CLO	93	64	53	36	146	1.8:1
Right side CLO	37	67	18	33	55	2.0:1
Left side CLO	49	60	33	40	82	1.5:1
CLO	7	80	2	20	9	3.5:1
CL ± P	251	71	103	29	354	2.4:1
BCL ± P	57	79	15	21	72	3.8:1
Cleft alveolus	201	72	80	28	281	2.5:1

Syndromic	41	53	37	47	78	1.1:1

Other congenital deformities	68	57	52	43	120	1.3:1
Congenital heart diseases	16	59	11	41	27	1.5:1
Dysmorphism	27	54	23	46	50	1.2:1
Central nervous system	6	67	3	33	9	2:1
Muscle deformities	6	75	2	25	8	3:1
Psychometric inadequacy	53	67	25	33	78	2:1
Total OFC	343	62	208	38	551	1.6:1

OFC, orofacial cleft; CL ± P, cleft lip and/or palate; CLO, cleft lip only; CLP, cleft lip and palate; CPO, cleft palate only; BCL ± P, bilateral cleft lip and/or palate.

**Table 2:** Dental caries among 6-year-old children with OFC and the comparable group from the general population in Norway.

Group	Total number	Number with caries	Prevalence (%)	*p*-value
OFC	551	99	18.0	0.891
General population	32,971	7,332	18.2	

**Table 3:** Association between caries and the different OFC, syndromic, and other congenital deformities.

Sub-group	*N*	Caries	
		OR (95% CI)	*p*-value
Girls	33	1 (ref)	
Boys	66	1.26 (0.79–1.99)	0.318

CPO	39	1 (ref)	0.627
CLO	23	0.76 (0.43–1.34)	0.337
CLP	37	0.88 (0.53–1.44)	0.606

UCL ± P	90	1 (ref)	
BCL ± P	9	0.62 (0.30–1.29)	0.195

No cleft alveolus	51	1 (ref)	
Cleft alveolus	48	0.89 (0.57–1.37)	0.581

Non-syndromic	83	1 (ref)	
Syndromic	16	1.21 (0.67–2.21)	0.528

No other congenital deformities	66	1 (ref)	
Other congenital deformities	33	2.10 (1.30–3.39)	0.002

No congenital heart disease	91	1 (ref)	
Congenital heart diseases	8	2.00 (0.85–4.72)	0.106

No dysmorphic trait	87	1 (ref)	
Dysmorphism	12	1.50 (0.76–2.10)	0.244

Psychometrically adequate	81	1 (ref)	
Psychometrically inadequate	18	1.45 (0.81–2.59)	0.207

Total	99		

CLO, cleft lip only; CLP, cleft lip and palate; CPO, cleft palate only; UCL ± P, unilateral cleft lip and/or palate; BCL ± P, bilateral cleft lip and/or palate; *N*, number; ref, reference category.

## 4 Discussion

OFC is the most common congenital deformity in the head and neck region, with an adverse effect on the child’s life (2–6). Dental caries is the most common infectious disease in humans (31). Hence, children with OFC may have more challenges in the oral cavity than others. The present study investigates children with OFC in Norway and the association between dental caries and different types of OFC, taking sex and other additional congenital deformities into consideration. To the best of our knowledge, this is the first study that examines the dental caries experience among 6-year-old children with OFC in Norway. The study utilized data obtained from the NRCLP.

The results from two studies in Sweden and Denmark showed that cleft lip with or without palate (CL ± P) constitutes approximately two-thirds of all OFC, whereas CPO represents the remaining one-third (32, 33), agreeing with the results from this study and the widely recognized nearly 2:1 distribution pattern (30). Some studies have identified CLP as the most prevalent form of OFC, followed by CPO, with CLO being the least common (34, 35). These findings align with our results, although the difference in prevalence between CLP and CPO in our study was relatively minor (36). Previous studies in Norway showed that CPO is the most common type of OFC (37, 38), as did results from studies in Northern Finland (39) and the UK (40). Hence, there is a variation among studies regarding which cleft type is the most common. This might be attributed to the population studied, the data available at the time of the study, and the inclusion and exclusion criteria (41).

Considering the sex distribution among children with OFC, evidence showed that there is an overall boy predominance (42). This agrees with our results and previous findings from Norway (37). Moreover, results from studies in Denmark and Iran showed the same male predominance (43, 44). In a study in Finland, girls were predominant (39).

Our study showed that boys were more common among CLO and CLP, while girls were more common among the CPO group. These results are compatible with results from China (36), and previous results from Norway (9, 37). Results from the UK (40), Finland (39), and Iran (44) showed similar sex distributions. A study from the USA showed a higher percentage of girls among the CPO children (45). In Norway, there is a predominance of males among children with cleft lip and palate compared with children with CLO (46). Our study showed a significant association between the cleft type and sex, which agrees with others (47).

A Swedish study showed a difference in caries prevalence between children with OFC and those without OFC (48). In addition, several studies (18, 49, 50) showed a higher prevalence of dental caries among children with OFC. Initially, we assumed that children with OFC in Norway at age 6 might have more caries than the comparable group from the general population. However, compared with national data for caries status of 5-year-old Norwegian children, which showed 18.2% (51), our results match. This suggests that Norway's follow-up dental programs for children with OFC may effectively offset structural and anatomical risk factors for dental caries identified among this group. The follow-up system of children with OFC in Norway starts from birth. They all participate in a national cleft care program. This includes follow-up at regional cleft centers, where they are regularly seen by a range of specialists. Oral health is an integrated part of this, including dental professionals from the national centers of cleft lip and palate as part of the team. Children with OFC typically get earlier, and regular, dental visits. In addition, their families are guided on oral hygiene, feeding, and other habits from birth. It may be this combination of structured care and continuous follow-up that reduces the risk of early caries, despite the anatomical and structural challenges within the oral cavity. Furthermore, the financial burden of dental visits, which is a significant burden of dental care, might be diminished because of the free dental treatment that all children have until the age of 18. In our study, the group of children with CPO had a slightly, but not statistically significant, increased caries experience. This agrees with others reporting no association between dental caries and OFC type (52). However, some studies have demonstrated significant associations between dental caries and OFC type (53, 54). This again suggests that a well-structured, individualized, regular dental visit and follow-up system, in addition to parental involvement in home-based oral hygiene, could overcome the anatomical challenges among different OFC types. In Norway, all children will (as a rule) get free, regular, and full dental care in the public dental health service. First dental visit starts at 3 years old and continues annually. Before 3 years old, parents get preventive instructions from primary care personnel. Early contact with the public dental service is made if needed (55). Children with OFC are enrolled early in the follow-up program. It is coordinated by the cleft lip and palate team in Oslo and Bergen, and the public dental health services (Public Dental Health Service). Individualized recall intervals for each child are scheduled according to the severity of the condition. High-level education of the parents in Norway (55) and the cooperation with the CLP team play an important role in children's oral health maintenance. Socioeconomic status and individual information about the number of dental visits and missed appointments are reported in each clinic's electronic patient journal (EPJ) and other national registries. These factors might have played a role in the overall oral health of children. Future studies linking NRCLP data to other national registries (e.g., Public Dental Health Service) are important to study the association with these possible factors.

An association was found between caries and other additional congenital anomalies. When cleft was combined with other additional congenital deformities, the caries experience was noticeably increased. Children with additional congenital anomalies may drop appointments, or dental care may be less important in the bigger picture of their medical needs. Furthermore, medications and eating, motor, or cognitive challenges may affect their oral hygiene in addition to cooperation challenges. The data on additional congenital deformities, dysmorphic features, and age-appropriate psychomotor development were mainly based on clinical evaluation by the plastic surgeon. Nevertheless, it showed an association with dental caries. Further studies to investigate the association between dental caries and other additional congenital deformities associated with children with OFC are needed.

As children with OFC have a higher risk of missing teeth in the permanent dentition (26), protection of the primary teeth through a well-structured health system and individualized treatment plans is important. More studies of oral health and caries status of primary teeth among children with OFC are needed to facilitate better-tailored support for the children.

## 5 Limitations

The present study used a dichotomized DMFT scoring. Furthermore, information about dental caries was based on clinical, OPG, and periapical x-rays. Bitewing x-ray should be considered for future studies. Due to the small subgroup, some analyses have low statistical power, leading to wide CI. The interpretations of these results must be performed with care.

## 6 Conclusion

Among Norwegian children, cleft lip and palate are more prevalent in boys than in girls. There was no difference in risk for caries between the different types of OFC, nor was there an increased risk compared with children in the same age group. However, children with OFC and additional congenital deformities have an increased risk of caries experience than children without such additional deformities. This indicates that more targeted, individualized, preventive oral health strategies are needed among this group.

## Data Availability

The data analyzed in this study are subject to the following licenses/restrictions: The data analyzed in this study were obtained from the NRCLP. Requests to access these datasets should be directed to lkg-registeret@helse-bergen.no.
